# Inhibition of the precursor and mature forms of HIV-1 protease as a tool for drug evaluation

**DOI:** 10.1038/s41598-018-28638-w

**Published:** 2018-07-11

**Authors:** Jana Humpolíčková, Jan Weber, Jana Starková, Eva Mašínová, Jana Günterová, Iva Flaisigová, Jan Konvalinka, Taťána Majerová

**Affiliations:** 1 Institute of Organic Chemistry and Biochemistry of the Czech Academy of Sciences, Flemingovo nám. 2, 16610, Prague 6, Czech Republic; 2 Department of Biochemistry, Faculty of Science, Charles University in Prague, 12843, Prague, Czech Republic

## Abstract

HIV-1 protease (PR) is a homodimeric enzyme that is autocatalytically cleaved from the Gag-Pol precursor. Known PR inhibitors bind the mature enzyme several orders of magnitude more strongly than the PR precursor. Inhibition of PR at the precursor level, however, may stop the process at its rate-limiting step before the proteolytic cascade is initiated. Due to its structural heterogeneity, limited solubility and autoprocessing, the PR precursor is difficult to access by classical methods, and limited knowledge regarding precursor inhibition is available. Here, we describe a cell-based assay addressing precursor inhibition. We used a reporter molecule containing the transframe (TFP) and p6* peptides, PR, and N-terminal fragment of reverse transcriptase flanked by the fluorescent proteins mCherry and EGFP on its N- and C- termini, respectively. The level of FRET between EGFP and mCherry indicates the amount of unprocessed reporter, allowing specific monitoring of precursor inhibition. The inhibition can be quantified by flow cytometry. Additionally, two microscopy techniques confirmed that the reporter remains unprocessed within individual cells upon inhibition. We tested darunavir, atazanavir and nelfinavir and their combinations against wild-type PR. Shedding light on an inhibitor’s ability to act on non-mature forms of PR may aid novel strategies for next-generation drug design.

## Introduction

Extensive studies of HIV-1 protease (PR) have expanded knowledge about the biological, chemical and structural aspects governing retroviral infections and led to successful development of antiretroviral drugs^1,2^.

To date, 10 PR inhibitors (PIs) have been approved by the Food and Drug Administration. The design of the more recently approved PIs in clinical use (particularly tipranavir, atazanavir and darunavir) was inspired by the effort to target drug-resistant PR variants^3,4^. However, targeting multidrug-resistant PR variants remains challenging^5^.

**Figure 1:**
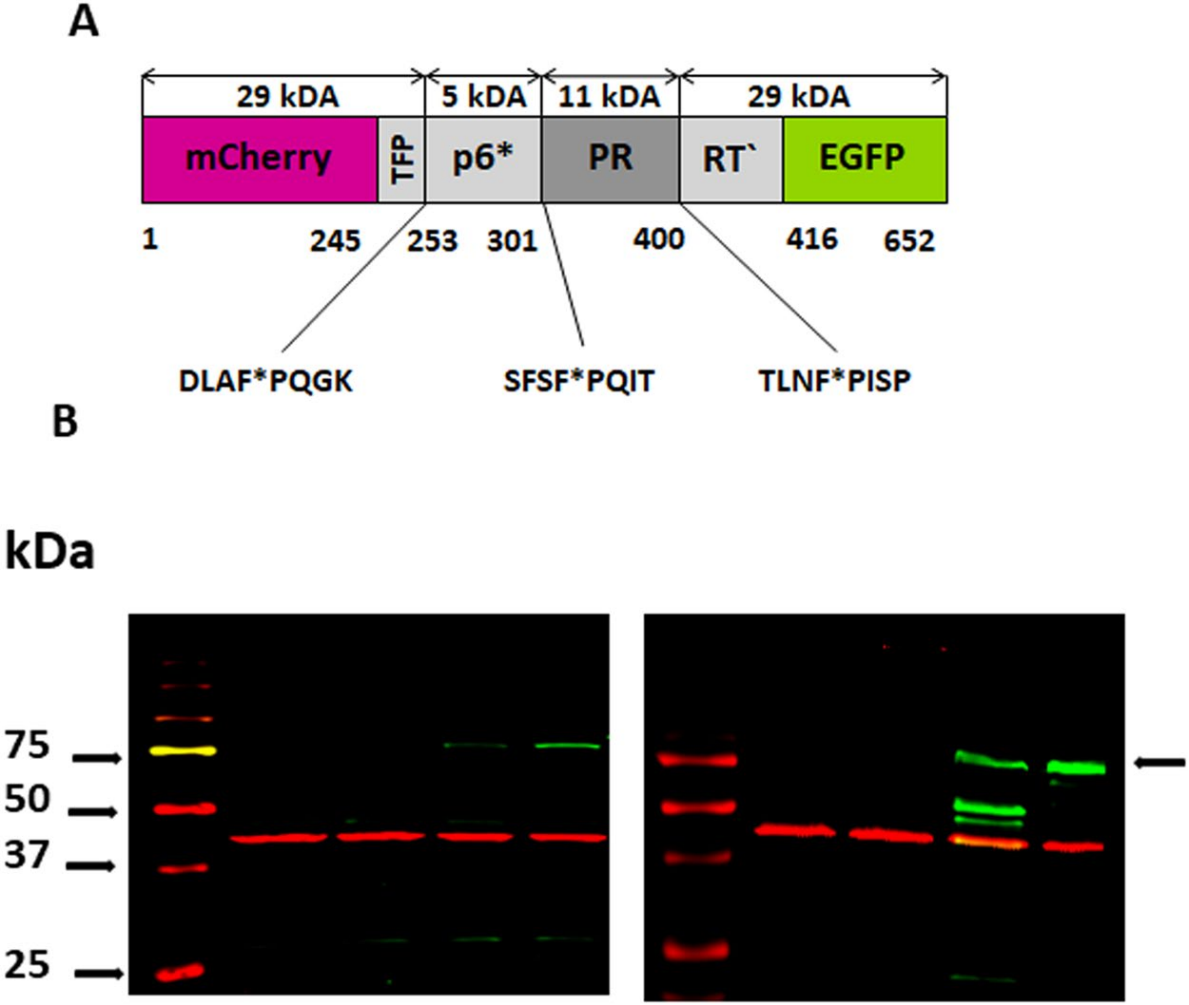
(**A**) Schematic representation of the uncleaved mCherry-PR*prec*-EGFP fusion protein: Two fluorescent proteins, mCherry and EGFP, are linked by an artificial HIV-1 PR precursor containing HIV-1 transframe peptide (TFP), p6* peptide, an HIV-1 PR monomer and a N-terminal fragment of reverse transcriptase. HIV-1 PR cleavage sites are marked with asterisks. (**B**) LEFT: Western blot analysis of HEK293T cell lysates detected with anti-mCherry antibody (green) (the arrow denotes the uncleaved precursor): Lanes: *1*. Marker *2*. Untransfected cells 3. Cells transfected with reporter harboring wild type PR without inhibitor. *4*. Cells transfected with reporter harboring PR treated with 80 nM darunavir. *5*. Cells transfected with reporter harboring wild type PR treated with 10 μM darunavir. RIGHT: Western blot analysis of cell lysates detected with anti-GFP antibody (green) – Lanes are the same as in the left panel. Detection of β-actin was used as a loading control (red).

HIV-1 PR is an obligatory homodimer, with each monomer contributing half of the active site. HIV-1 PR is produced as part of the Gag-Pol polyprotein. It is encoded in the Pol region and is flanked by p6* peptide at its N-terminus and reverse transcriptase at its C-terminus. Each Gag-Pol polyprotein contains one HIV-1 PR monomer (Fig. 1A). HIV-1 PR autoproteolysis is a key step in viral maturation, which is critical for successful production of infectious viral progeny^1^.

Autoprocessing depends on transient dimer formation followed by intramolecular (*cis*) cleavage of the PR precursor. As suggested by *in vitro* studies, the first *cis* cleavage event does not occur directly adjacent to termini of PR. Instead, one site in the Gag region (p2-NC) and one site in the Pol region (TFP-p6*) are cleaved intramolecularly, followed by *cis* N-terminal cleavage of HIV-1 PR out of the precursor. The remaining cleavage sites are processed intermolecularly (*trans* cleavage)^6–8^.

Inhibition of HIV-1 PR leads to production of immature non-infectious viral particles^1^, but it is not the only PR-related mechanism that can hamper the virus. A delay in HIV-1 autoprocessing leads to formation of viral particles with irregular morphology^9^, while overactivation of HIV-1 PR blocks production of viral progeny^10,11^.

Clearly, the activation and activity of HIV-1 PR must be perfectly orchestrated. However, the details of these processes remain poorly understood^12^. Studies have shown that the PR precursor has a much lower tendency to form dimers than mature PR^13,14^, and it shows much lower activity and possibly modified specificity^15–17^. On the other hand, it is likely stabilized by substrate binding^18^. Viral p6* protein, located directly upstream of the PR domain (Fig. 1A), prevents premature PR activation. Four C-terminal p6* residues appear to be indispensable for this function^19^, analogous to zymogenic forms of monomeric aspartic proteases^20–25^.

All PR inhibitors in clinical use target the active site (although a possible secondary binding site has been reported for tiprinavir and darunavir^26–28^) and bind the PR precursor several orders of magnitude less strongly than mature PR^6,17,29–31^. However, compounds targeting the PR precursor could be attractive drug candidates^32–34^.

Although there are hundreds of available X-ray structures of mature PR free or in complex with different inhibitors, little is known about the structure of the PR precursor. Predictions of intrinsic disorder revealed an almost unstructured p6* region and disordered flap region^35^. This flexibility may enable the existence of an equilibrium of conformations^36^, dynamically shifting in response to changes in conditions such as packaging into viral particle, proteolysis and ligand binding. NMR studies with an artificial precursor revealed that embedded PR comprises a population of partially folded species, and only a small portion is able to form dimers^37^. High-resolution crystal structures of a model PR precursor possessing four C-terminal amino acids of the p6* peptide in complex with darunavir or saquinavir revealed that the sequence flanking mature PR is conformationally variable. However, the binding mode and conformation of the active site did not differ significantly from the mature structures^38^. This could be a result of the inhibitor’s propensity to bind a mature-like subpopulation most tightly and shift the equilibrium in this direction during crystal growth.

Because the structure of the precursor likely is flexible, ligand-based drug design^39,40^ in combination with high-throughput screening may be a promising approach to target PR in its precursor form.

We developed a cell-based assay to assess inhibition of autoprocessing. The assay exploits Förster resonance energy transfer (FRET) between two fluorescent proteins, EGFP and mCherry, as well as the cytotoxicity of PR. We constructed a reporter in which PR and its natural flanking sequences, containing the first *cis*-cleaved TFP-p6* site, is inserted between mCherry and EGFP (Fig. 1A). When cells are transiently transfected and express the reporter, the toxicity of HIV-1 PR^41–43^ results in loss of fluorescence. Addition of a PR-specific inhibitor restores the fluorescence. To distinguish whether the inhibition occurred due to inhibition of the precursor or mature PR, we measured the level of FRET between EGFP and mCherry. In the case of inhibition of mature PR, mCherry and EGFP are present as separate entities, and no FRET is observed. On the other hand, inhibition of the mCherry-PR-EGFP precursor (Fig. 1A) keeps these two fluorescent molecules in proximity to each other and enables FRET.

To justify our assumptions about the behavior of the reporter in cells, we also employed two independent microscopy assays to evaluate reporter autoprocessing: (i) fluorescence cross-correlation spectroscopy (FCCS), a single-molecule technique that monitors the mutual motion of EGFP and mCherry, and (ii) fluorescence life-time imaging (FLIM) in combination with FRET, which monitors the proximity of EGFP and mCherry directly in individual cells. The presence of FRET is determined by a decrease in donor fluorescence lifetime. Furthermore, we employed intensity-based FRET in a flow cytometry assay to precisely quantify the precursor inhibition.

Taking the examples of darunavir, atazanavir and nelfinavir interacting with wild-type PR, we showed that PR forms differ in their affinity to these compounds. We compared these results with findings from a drug susceptibility assay with fully infectious virus. Our data suggest synergy between darunavir and atazanavir and antagonism between atazanavir and nelfinavir. The synergistic effect could be explained by different modes of interaction of the inhibitors with various forms of PR (differing in length and/or conformation).

Our assay also can work in a high-throughput setup to screen compounds of interest and help identify a set of ligands targeting the PR precursor, which can become a starting point for ligand-based design of additional compounds.

## Results

### The reporter harboring an artificial PR precursor undergoes autoprocessing

**Figure 2:**
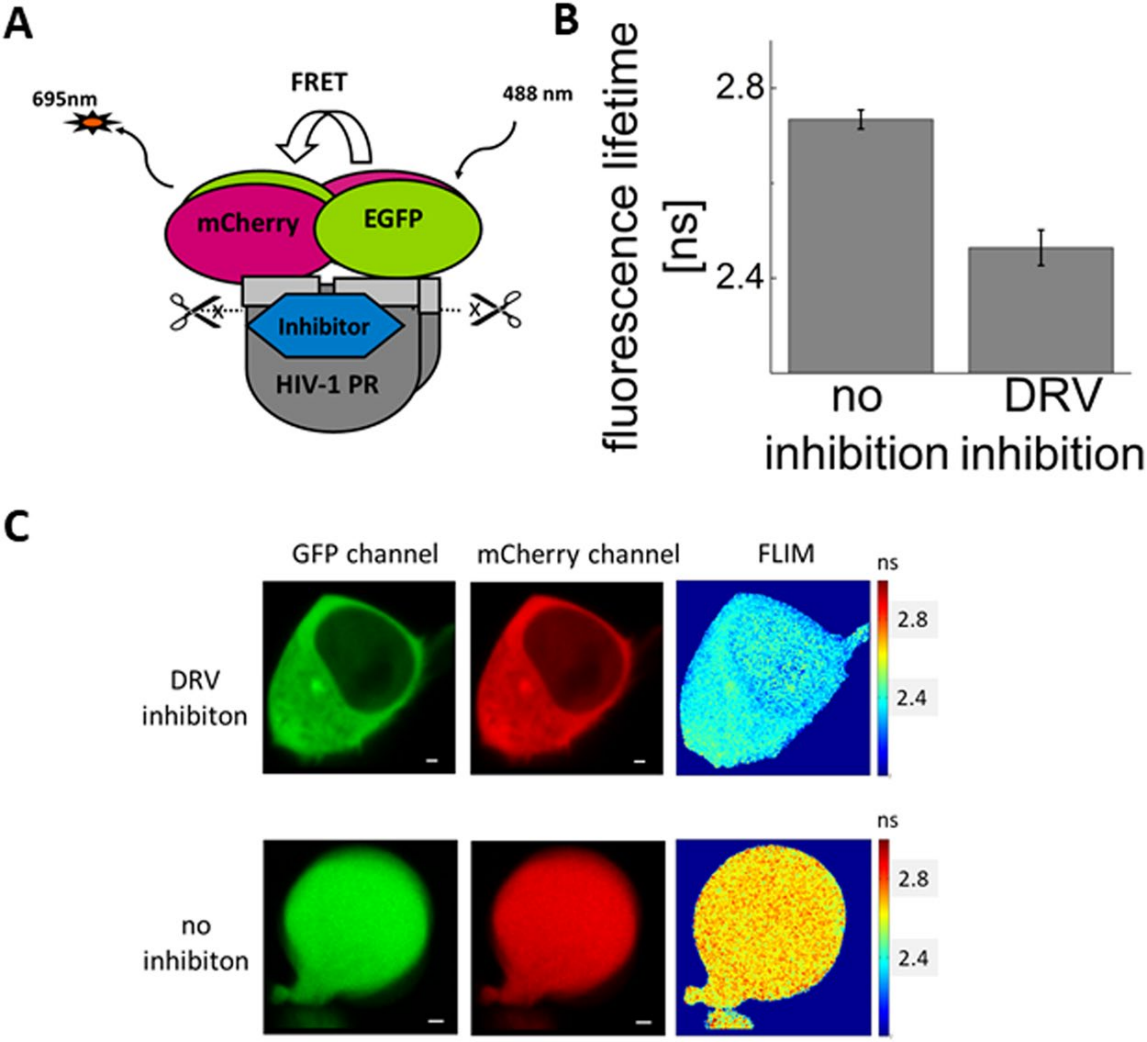
Fluorescent life-time imaging (FLIM) indicates FRET between EGFP and mCherry in the mCherry-PR*prec*-GFP precursor. (**A**) Schematic representation of the autoprocessing inhibition assay based on expression of the mCherry-PR*prec*-GFP reporter. (**B**) Quantification of the FRET level based on life-time comparison of 1 μM darunavir (DRV)-treated and untreated cells (**C**). Fluorescent images of HEK293T cells transfected with the mCherry-PR*prec*-EGFP reporter inhibited/non-inhibited with 1μM DRV: green and red channels and a fluorescent life-time image. The scale bar indicates 5 µm. The error bar represents the standard error of the mean. The FLIM experiment was performed for 10 cells in both conditions.

A PR precursor containing TFP-p6*, p6*-PR and PR-RT cleavage sites was inserted between the mCherry and EGFP fluorescent proteins to obtain mCherry-TFP-p6*-PR-RT′-EGFP (denoted mCherry-PR*prec*-EGFP, Figs 1A and 2A).

When expressed in transiently transfected mammalian cells, the reporter retained its autoprocessing ability and cytotoxicity. Expression and autoproteolysis of the reporter was confirmed by Western blot using anti-mCherry and anti-EGFP antibodies (Fig. 1B). Due to the cytotoxicity of PR, the expression of the reporter was very low. However, it dramatically increased upon addition of a PI. At lower inhibitor concentrations, the cleavage products were detectable; at higher inhibitor concentrations, autoprocessing was impaired and the uncleaved precursor appeared.

### Reporter autoprocessing can be visualized by fluorescence microscopy

**Figure 3:**
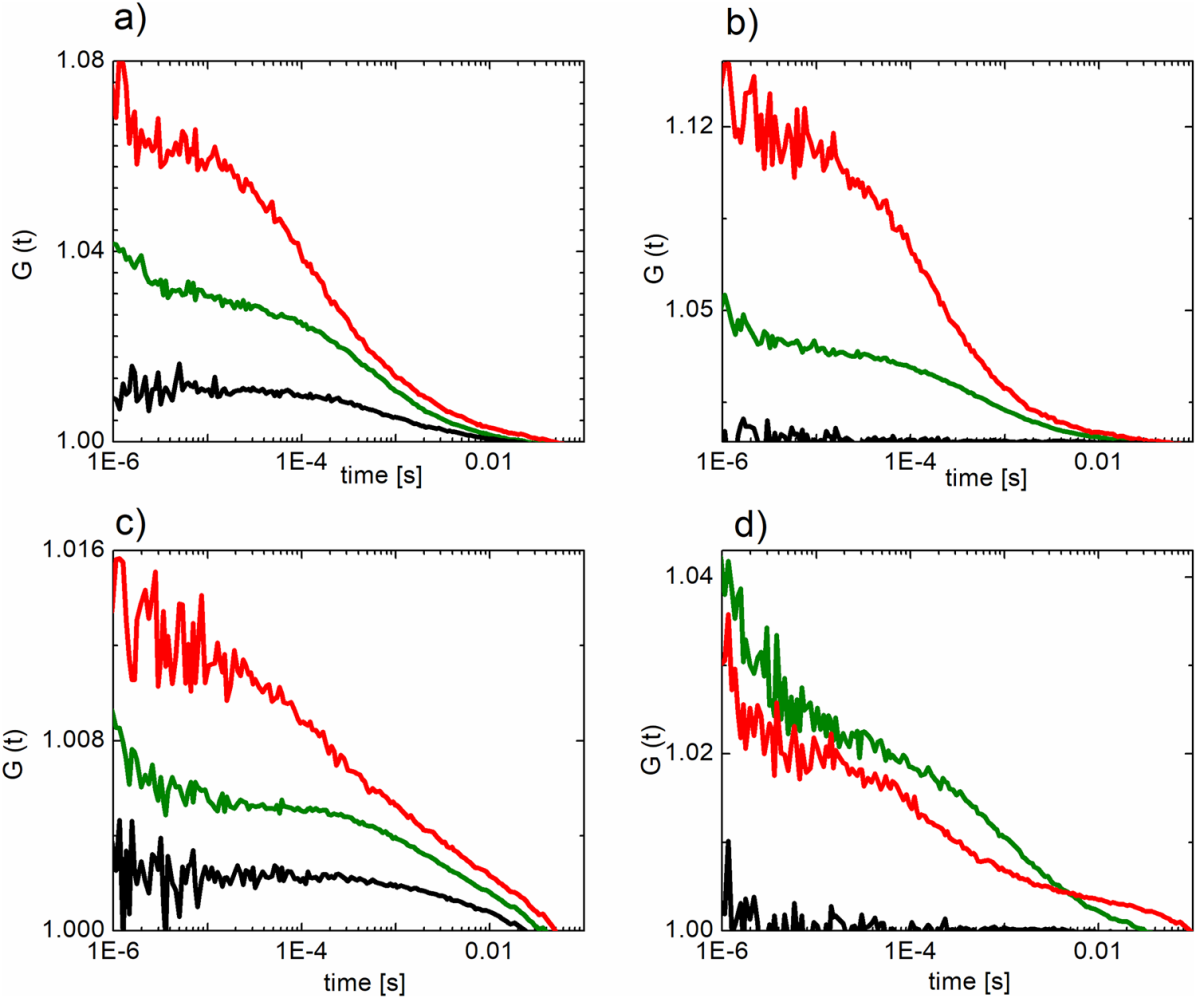
Fluorescence cross-correlation spectroscopy (FCCS) experiments were performed in HEK293T cells transiently transfected with: (**a**) pmCherry-EGFP fusion as a positive control, (**b**) pEGFP-N1 and pmCherry-C1 as a negative control, (**c**) mCherry-PR*prec*-EGFP reporter inhibited with 1μM darunavir, (**d**) mCherry-PR*prec*-EGFP (non-inhibited). Autocorrelation functions of the EGFP and mCherry fluorescent signal are shown in green and red, respectively. The crosscorrelation function is shown in black.

We detected the PI-dependent accumulation of the mCherry-PR*prec*-EGFP fusion in treated cells by fluorescence cross-correlation between EGFP and mCherry using confocal microscopy. The reporter (unless autoprocessed) contains both fluorescent proteins moving simultaneously. Using fluorescence cross-correlation spectroscopy (FCCS), a single-molecule technique, we calculated the temporal cross-correlation function (CCF) between fluorescence fluctuations in the EGFP and mCherry detection channels. The fluctuations are caused by individual molecular transits through the overlapping *foci* of the two excitation laser beams. Therefore, if the two molecules move together, the fluctuations are correlated, which translates to increased amplitude of CCF (relative to the amplitudes of individual autocorrelation functions – ACFs; Fig. 3c). As a positive control, we used fused mCherry and EGFP, which gave a positive CCF amplitude (Fig. 3a), while our negative control—cells transiently expressing individual EGFP and mCherry—gave a flat CCF profile (Fig. 3b). When the reporter is autoprocessed, the two fluorophores start moving individually, resulting in a flattened CCF (Fig. 3d). We confirmed that in the absence of a PI, the two fluorophores move independently, whereas treatment of cells with 1 μM darunavir led to an increase in the amplitude of the CCF, indicating that mCherry and EGFP remained fused.

As a single-molecule approach, FCCS can only be used at relatively low expression levels. At high expression levels, the signal fluctuations are small compared to the overall signal and thus other techniques have to be employed. We selected cells with lower levels of expression for the analysis.

To assess the possibility of FRET between EGFP and mCherry in the mCherry-PR*prec*-EGFP fusion, we used fluorescence life-time imaging (FLIM). In contrast to FCCS, FLIM-FRET is a microscopy technique that can reveal reporter autoprocessing based on the spectroscopic properties of EGFP (interacting with mCherry) rather than by analyzing the molecular dynamics. It can be applied even in cells that highly express the reporter and enables sensing of molecular vicinity in the nanometer range. Therefore, the presence and absence of FRET indicates non-processed and processed reporter molecules, respectively. In the mature PR dimer, the N- and C-termini are close together, forming the dimerization domain. We expected that the distance between the N- and C-terminal parts of our artificial precursor may enable FRET between the N-terminal mCherry and C-terminal EGFP. Indeed, in PI-treated (1 μM darunavir) cells, we measured a marked decrease in fluorescence lifetime using confocal microscopy upon excitation at 490 nm, corresponding to EGFP quenching (Fig. 2).

### Quantification of reporter autoprocessing reveals differences in inhibitor binding to PR precursor

**Figure 4:**
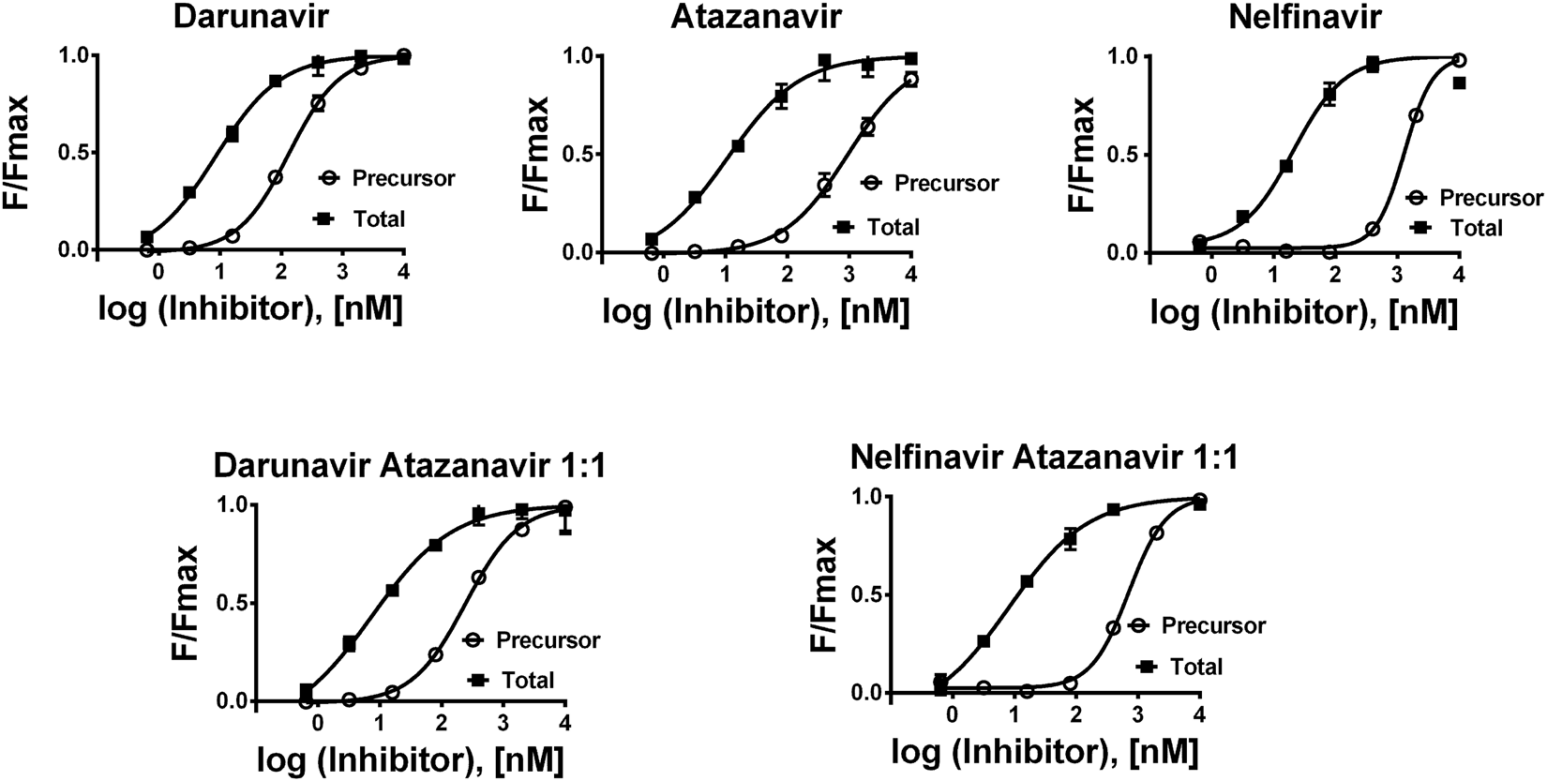
Dose response curves: Flow cytometry analysis of HEK293T cells transiently transfected with the mCherry-PR*prec*-EGFP reporter harboring wild-type HIV-1 protease and treated with an appropriate concentration of inhibitor(s). In the case of PI mixtures, the ratio of inhibitors was 1:1. The mCherry signal (measured at 610 nm upon excitation at 561 nm) corresponds to the total inhibition of all PR forms (precursor, partially cleaved precursor and mature form), whereas the FRET signal (measured at 695 nm upon excitation at 488 nm) corresponds to the amount of PR precursor. The mean fluorescence (F) for the appropriate inhibitor concentration was divided by the maximal reachable mean fluorescence (Fmax) of the transfected cells in the presence of the appropriate inhibitor.

We next used flow cytometry to investigate the signals of individual fluorophores and FRET between them in HEK293T cells transiently transfected with mCherry-PR*prec*-EGFP and treated with PIs. Flow cytometry analysis revealed dose-dependent increases in fluorescence in the presence of clinically used PIs in both the mCherry (561 nm/610 nm) and EGFP (488 nm/530 nm) channels. The FRET signal of mCherry-PR*prec*-EGFP (488 nm/695 nm) dramatically increased at higher inhibitor concentrations (Fig. 4). The increase in fluorescence results from the inhibition of PR and consequent blocking of its cytotoxicity.

The mCherry signal is independent of the presence of FRET. Therefore, the mCherry signal reports on the total inhibition of all the PR variants (precursor, partially processed precursor, mature form), whereas the intensity of the FRET signal specifically quantifies the amount of unprocessed precursor.

All the inhibitors tested have markedly lower affinity to the precursor than to the mature form (Fig. 4, Table 1). Darunavir retained good inhibitory potency against the PR precursor. Although the total inhibition of atazanavir is comparable to that of darunavir, its affinity to the precursor was markedly weaker. On the other hand, nelfinavir has a weaker affinity to all PR forms. However, its affinity to the PR precursor was comparable to that of atazanavir. The ratio of its half-maximal effective concentration (EC_50_) for the precursor to total PR inhibition was higher than that of atazanavir. These differences in the propensities of the PR forms to bind an inhibitor indicate possible different modes of interaction between the individual inhibitors and different forms of PR (Fig. 4, Table 1).

**Table 1:** EC_50_ values obtained using two independent assays.

Drug(s)	Total inhibition of HIV-1 PR (EC_50_, nM)	Inhibition of HIV-1 PR precursor (EC_50_, nM)	Antiviral activity (EC_50_, nM)
Darunavir	8.5 ± 1.5	133 ± 7	4.3 ± 0.5
Atazanavir	10.8 ± 3.6	937 ± 171	1.4 ± 0.1
Nelfinavir	21 ± 6	1302 ± 59	10.8 ± 1.0
Darunavir + Atazanavir 1:1	7.3 ± 3.6	242 ± 32	0.7 ± 0.1
Nelfinavir + Atazanavir 1:1	8.2 ± 2.3	706 ± 44	0.5 ± 0.1

The total PR inhibition and precursor PR inhibition were quantified using HEK293T cells transiently transfected with the mCherry-PR*prec*-EGFP reporter. In parallel, antiviral activity was determined with MT4 cell-based CPE protection assay with NL4-3 virus. The error was expressed as a standard error.

In parallel, we performed a drug susceptibility assay with the fully infectious NL4-3 virus. This confirmed the lower inhibitory activity of nelfinavir (Table 1).

**Figure 5:**
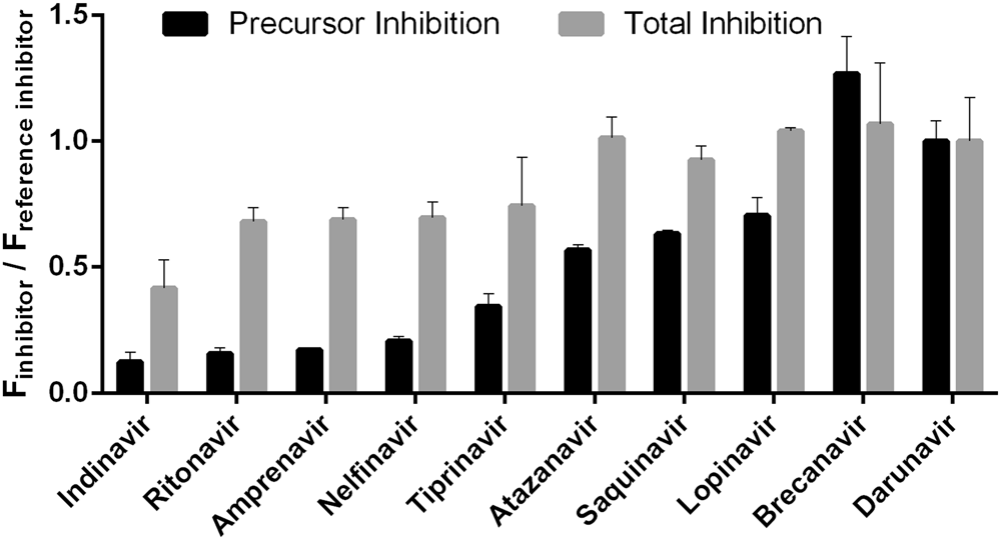
Screening of HIV-1 protease inhibitors: HEK293T cells were transiently transfected with the mCherry-PR*prec*-EGFP reporter and treated with 400 nM of each inhibitor in triplicate. Darunavir was used as a reference compound. After 24 h, the cells were harvested and analyzed by flow cytometry. The mCherry signal corresponds to the total inhibition of all PR forms (precursor, partially cleaved precursor and mature form), whereas the FRET signal corresponds to the amount of PR precursor. The mean fluorescence (F) for the appropriate inhibitor was divided by the fluorescence (F) obtained for darunavir. The error was expressed as a standard error.

In addition, we screened a set of FDA-approved PIs and one experimental compound (brecanavir). We used a fixed inhibitor concentration of 400 nM, at which we expected to observe significant differences in precursor inhibition. Darunavir was used as a reference. The recently developed compounds blocked precursor processing more efficiently than the older drugs (Fig. 5). However, the oldest PI, saquinavir, which received FDA approval in 1995, inhibited the precursor at levels comparable to newer PIs.

### Darunavir and atazanavir act synergistically

We also investigated possible synergy/antagonism between darunavir and atazanavir and between atazanavir and nelfinavir. Because these phenomena are not straightforward to recognize from EC_50_ values (Table 1), we analyzed the data (inhibitor dose vs. response) with CompuSyn software, which uses the Chou-Talay method based on a median-effect equation to calculate combination indexes (CI). This method is mechanism-independent and takes into account not only the potency of inhibitors and their mixtures, but also the shape of the dose-response curve^44^. We identified weak synergy between darunavir and atazanavir (CI < 1) and antagonism between nelfinavir and atazanavir (CI > 1) in cells transiently transfected with the mCherry-PR*prec*-EGFP reporter, as well in the drug susceptibility assay with fully infectious virus (Table 2).

**Table 2:** Combination indexes (CI) were calculated according to the Chou-Talay method from the data obtained from fluorescence vs. inhibitor(s) dose from the mCherry-PR*prec*-EGFP-based assay measured by flow cytometry and from the drug susceptibility assay with NL4-3 virus and the predominating effect (additive for CI = 1, synergism for CI < 1 and antagonism for CI > 1) is shown in bold.

Drug combination indexes CI in two independent assays	CI:EC_50_	CI:EC_75_	CI:EC_90_	CI:EC_95_	Result
DRV + ATV Virus	**0**.**72**	**0**.**55**	**0**.**42**	**0**.**36**	**Synergism**
DRV + ATV Total PR inhibition	1.05	0.93	**0**.**84**	**0**.**79**	Additive/**Synergism**
DRV + ATV Precursor inhibition	1.03	1.05	1.07	1.08	**Additive**
ATV + NFV Virus	0.63	**1**.**19**	**2**.**38**	**3**.**93**	**Antagonism**
ATV + NFV Total PR inhibition	**1**.**27**	**4**.**20**	**15**.**9**	**41**.**9**	**Antagonism**
ATV + NFV Precursor inhibition	**1**.**43**	**1**.**31**	**1**.**20**	1.13	**Antagonism**

## Discussion

We constructed a reporter harboring the fluorescent proteins mCherry and EGFP, separated by an artificial HIV-1 PR precursor (Figs 1A, 2A). This precursor was derived from the viral Pol polyprotein and contains the full-length TFP, p6* peptide, a PR monomer and 16 amino acids from the N-terminus of reverse transcriptase. The precursor contains two sites anticipated to be cleaved in *cis* from the Gag-Pol precursor. The first cleavage site lies between the TFP and p6* peptide and the second between p6* peptide and the N-terminus of PR. The third cleavage site in our reporter lies between the PR C-terminus and the N-terminus of reverse transcriptase and undergoes *trans* cleavage (6–8).

When cells transfected with the reporter were treated with a PI, the dose-dependent accumulation of unprocessed precursor was detectable by Western blot (Fig. 1B), by an increase in cross-correlation between fluorophores (mCherry and EGFP measured by FCCS, Fig. 3), by a decrease in the fluorescence lifetime of EGFP (measured by FLIM, Fig. 2) and by an increase in the FRET signal measured by flow cytometry. We used the latter feature to quantify the chimeric precursor in cells upon treatment with inhibitors (Fig. 4, Table 1).

Cells transfected with the reporter and untreated with inhibitor had much lower fluorescence than inhibitor-treated cells, due to PR toxicity^41,43^. Differences in cytotoxicity also should be taken into an account when several PR variants are tested against the same panel of inhibitors. In such cases, one of the inhibitors (ideally a strong binder) should be used as a reference compound.

Quantification of the inhibitor dose-dependent accumulation of the precursor by FRET between mCherry and EGFP is not dependent on the cytotoxicity and can be applied to nontoxic PR variants. Problems with precise determination of EC_50_ *via* dose-dependent quantification of the FRET signal can arise in cases in which the EC_50_ is very high and saturation with the inhibitor is not reachable due to its toxicity, limited solubility or biological availability. Still, comparison of the affinities of individual inhibitors to the precursor is possible at a fixed concentration. An example of such an experimental setup is shown in Fig. 5. For high-throughput screening applications, choice of an appropriate inhibitor concentration is important and depends on the purpose of screening. For precursor inhibition, we used concentrations one to two orders of magnitude higher than for screening of total PR inhibition.

We tested the effects of darunavir, atazanavir and nelfinavir on this reporter (Fig. 4, Table 1). In all cases, the inhibitors had an EC_50_ for precursor inhibition markedly higher than for total inhibition, as expected based on previous work^6,29–31^. These previous studies evaluated autoprocessing by Western blot or SDS-PAGE of artificial precursors^32^ and showed that the affinities of PIs to the PR precursor are several orders of magnitude lower than their affinities to the mature form. This effect can be more pronounced in the case of PI-resistant mutants^30^. Moreover, PR mutations can have different impacts on the substrate specificity of mature PR and its corresponding precursor^17^.

Using our reporter (Fig. 1), we determined EC_50_ values for darunavir, atazanavir and nelfinavir. Darunavir was the most potent inhibitor for both variants and blocked both precursor variants in a comparable manner. Atazanavir was a potent inhibitor from the point of view of total inhibition; however, its ability to block both precursor variants was markedly weaker. Nelfinavir was the weakest inhibitor from the point of view of total inhibition, but its propensity to bind both precursors was comparable to that of atazanavir (Table 1, Fig. 4). The differences observed in the binding of individual inhibitors to the precursor and processed form(s) may indicate different modes of interaction. A potential alternative binding site blocking dimerization has been reported for darunavir^28^. This could be one reason why this drug displayed the best precursor inhibition (still one and half orders of magnitude weaker than inhibition of the processed form).

We next combined two inhibitors in our assay: darunavir with atazanavir and atazanavir with nelfinavir (Table 2). The data were processed according to Chou and Talay^45^ using an algorithm that takes into the account not only absolute values but also the shape of drug-response curve. The obtained combination indexes (CI) indicate synergy between darunavir and atazanavir and antagonism between atazanavir and nelfinavir. We verified these results using a drug susceptibility assay with fully infectious virus (Table 2).

Antagonism occurs when two ligands compete for the same binding site on the molecule of a receptor. This situation is expected in the case of two active-site targeted inhibitors. The interpretation of synergy is less obvious. In general, synergistic binding indicates that two different ligands can bind to one molecule of a receptor at the same time (being mutually non-exclusive). In the case of darunavir, its potential alternative binding site could play a role. However, binding of darunavir to this putative alternative site would block PR dimerization, preventing the formation of the active site. Thus, even taking this alternative mechanism into account, the two inhibitors would be mutually exclusive. Other investigators have reported synergy or additive effects between other PI combinations^46–48^. Although their results are not fully consistent (and were obtained using different cells with different viral variants), synergy between saquinavir and lopinavir or atazanavir for a drug-resistant mutant seems to be the most well-established. One possible explanation for this synergy is that one inhibitor can increase the intracellular concentration of another inhibitor through interactions with cellular transporters^47^.

Alternatively, we propose that synergy between PIs depends on the presence of different PR forms with various lengths (precursor, partially processed precursor, mature form), each representing a population of conformations—a conformational ensemble^44,49^. Liu *et al*. assessed the conformational landscape of mature PI-resistant variants by pulsed paramagnetic double electron-electron resonance (DEER). They found that in the presence of darunavir, the PR population is dominated by the closed conformation, whereas in the presence of atazanavir or nelfinavir, other conformations are preserved to some extent^50^. With regards to the disordered structure of p6*, the full-length and partially processed precursors can have quite wide conformational distributions. We suggest that the synergy/additive effect could result from binding of different inhibitors to different subpopulations of partially folded or intrinsically disordered and partially processed precursor variants. The inhibitor binding also could shift the distribution of equilibrium between all these forms.

Note that an additive effect at the precursor level may be enhanced at the mature PR level because precursor cleavage is the first event in the cascade and blocking of the precursor stops the subsequent “multiple domino effect”.

Although these interactions of inhibitors with PR precursors are likely too subtle to have a real clinical impact (precursor binding by darunavir is the most promising), stronger precursor binders could be attractive drug candidates. Such compounds would block the first step of the cascade of PR action, leading to no or markedly decreased production of mature, fully active PR. The residual portion of mature PR could be inhibited by classical inhibitors. This would create a double selection pressure on the virus, lowering the probability of selection of drug-resistant variants. Such combinations of a precursor inhibitor and mature form inhibitor also could enable reduced drug doses.

Our findings suggest that taking the intrinsic disorder of proteins into account may aid the development of next-generation drugs targeting various protein forms, including but not limited to PR and its precursor.

## Material and Methods

### Antiretroviral drugs, plasmid, cells and viruses

The following reagents were obtained through the NIH AIDS Reagent Program, Division of AIDS, NIAID, NIH: atazanavir sulfate, nelfinavir, amprenavir, saquinavir, indinavir, ritonavir lopinavir, tipranavir; darunavir from Tibotec, Inc.; MT-4 cells from Dr. Douglas Richman; pNL4-3 plasmid from Dr. Malcolm Martin. Brecanavir was obtained from GlaxoSmithKline. HEK293T cells were obtained from ATCC. MT-4 cells were maintained in RPMI1640 medium with L-glutamine supplemented with 10% fetal bovine serum (FBS), 100 U/ml penicillin, 100 µg/ml streptomycin and 10 mM HEPES (all Sigma-Aldrich). HEK293T cells were maintained in Dulbecco’s Modified Eagle’s Medium (DMEM) with L-glutamine, 10% FBS, 100 U/ml penicillin and 100 µg/ml streptomycin (all Sigma-Aldrich). HEK293T cells were transfected with pNL4-3 to produce NL4-3 virus, as previously described^51^. The tissue culture dose for 50% infectivity was determined in triplicate using the Reed and Muench method^52^.

#### Cloning of the reporter

Cloning was performed in three steps. First, the pmCherry-C1 vector was cleaved with *SacI* restriction enzyme (New England Biolabs), and the cohesive ends were blunted with T4 DNA polymerase (New England Biolabs) and ligated with T4 DNA ligase (New England Biolabs). Second, the resulting vector was cleaved with *HindIII* and *XbaI* restriction enzymes (New England Bioloabs). The same pair of restriction enzymes was used to generate the insert from the pEGFP-N1 plasmid, which contains the EGFP open reading frame. These two fragments were ligated. Third, the resulting mammalian expression vector containing the mCherry-polylinker-EGFP open reading frame was cleaved with *HindIII* and *KpnI* restriction enzymes (New England Biolabs). The HIV-1 protease precursor was obtained by PCR amplification from the pNL4-3 plasmid using the primer pair 5′-TTTAAGCTTTTTTTTAGGGAAGATCTGGCC-3′ and 5′-TTTTGGTACCGTCTTTAATTTTACTGGTACAGTC-3′. The PCR product was cleaved with *HindIII* and *KpnI* restriction endonucleases and ligated into the vector.

#### Transfections

HEK293T cells were grown overnight in Dulbecco’s Modified Eagle Medium [DMEM high glucose without L-glutamine with sodium pyruvate (Biosera)] supplemented with 10% fetal calf serum (Sigma Aldrich) and L-glutamine (Sigma Aldrich) to a final concentration of 4 mM. The cells were grown to 30–40% confluency. Prior to transfection, the mammalian expression plasmid (0.5 μg) was mixed with 25 μl Gibco™ Opti-MEM™ (SigmaAldrich) and 1.5 μl polyethylenimine, linear, MW 25,000 (Polysciences Inc) per well in a 24-well plate. (A 1 mg/ml polyethylenimine stock solution was prepared according to the manufacturer’s instructions.) This mixture was left at room temperature for 15 min and then added to the cell culture. Immediately after transfection, an appropriate concentration of an inhibitor in 1 μl DMSO was added. Cells were harvested after 24 h.

#### Western blotting

Harvested aliquots of 10^6^ HEK293T cells were lysed. Lysates were fractionated by SDS-PAGE and transferred onto Protran BA 85 nitrocellulose membranes (Schleicher & Schuell Keene, New Hampshire). The membranes were probed with the antibody of interest [GFP (D5.1) XP rabbit monoclonal or anti-red fluorescent protein rabbit polyclonal (BioRad)] and anti β-actin mouse monoclonal antibody (A5441, Sigma-Aldrich). IRDye® 800CW goat anti-rabbit IgG and IRDye® 680RD goat anti-mouse IgG (Li-Cor Biotechnology) were used as secondary antibodies. The Western blots were developed and detected with an Odyssey® CLx Imaging System according to the manufacturer’s instructions.

#### Confocal microscopy

Fluorescence cross-correlation spectroscopy (FCCS) and fluorescence lifetime imaging (FLIM) to address FRET were performed with an LSM 780 confocal microscope (Zeiss, Jena, Germany) equipped with the LSM upgrade kit (Picoquant, Berlin, Germany) enabling time-correlated single photon counting (TCSPC) acquisition. EGFP was excited by an InTune laser (Zeiss) at 490 nm wavelength and 40 MHz repetition frequency; mCherry was excited by a 561-nm solid state continuous wave laser. A 40x/1.2 water objective was used together with a pinhole in the detection plane. Behind the pinhole, the light was split on two tau-SPAD detectors (Picoquant) equipped with 525/45 and 600/52 band pass filters (Semrock, Rochester, NY) for EGFP and mCherry, respectively.

For FCCS, data were correlated with a Matlab (Mathworks, Natick, MA) script that was developed in-house according to a previously described algorithm^53^. To avoid detector crosstalk, the red channel fluorescence signal was split according to its fluorescence decay pattern (exponential for the signal generated by the pulsed InTune laser and flat for the 561-nm continuous wave laser) into two contributions, and only the signal assigned to the flat decay profile was correlated. Details of the data processing are given elsewhere^54^.

For FLIM-FRET, fluorescence decays in all pixels were transformed to the phasor diagram^55^ in which the prevailing population of pixels with identical decay function was selected. The overall decay from all the selected pixels was calculated, and integrated lifetime was used as a FLIM read-out:

1 $$\tau =\frac{\int t\,\ast \,I(t)dt}{\int I(t)dt}$$

#### Flow cytometry analysis of cells expressing the mCherry-PR*prec*-EGFP reporter

Expression of EGFP, mCherry and the mCherry- PR*prec* -EGFP fusion was detected 24 h after transfection using BD LSRF FoertessaTM flow cytometer (BD Biosciences) and processed with BD FACSDiva 8.0.1 software (BD Biosciences). The following channels were used: EGFP (488 nm/530 nm), mCherry (561 nm/610 nm), mCherry- PR*prec* -EGFP (488 nm/695 nm). The mean fluorescence of positive single alive cells was determined. The signal from nontransfected cells was subtracted. Due to the partial overlap of the mCherry- PR*prec* -EGFP signal with the remaining two signals, the following correction was performed:

2 $${{{\rm{F}}}_{{\rm{cor}}}}^{{\rm{m}}{\rm{C}}{\rm{h}}{\rm{e}}{\rm{r}}{\rm{r}}{\rm{y}} \mbox{-} {\rm{PR}}prec \mbox{-} \mathrm{EGFP}}={{{\rm{F}}}_{488/695}}^{\mathrm{mCherry} \mbox{-} \mathrm{PR}prec \mbox{-} \mathrm{EGFP}}\,-\,{{\rm{x}}}^{\ast }{{{\rm{F}}}_{488/530}}^{\mathrm{mCherry} \mbox{-} \mathrm{PR}prec \mbox{-} \mathrm{EGFP}}{-y}^{\ast }\,{{{\rm{F}}}_{561/610}}^{\mathrm{mCherry} \mbox{-} \mathrm{PR}prec \mbox{-} \mathrm{EGFP}}$$

where

3 $$x={{{\rm{F}}}_{488/695}}^{{\rm{EGFP}}}/{{{\rm{F}}}_{488/530}}^{{\rm{EGFP}}}$$

(obtained from cells transfected with pEGPP-N1),

4 $${\rm{y}}={{{\rm{F}}}_{488/695}}^{{\rm{mCherry}}}/{{{\rm{F}}}_{561/610}}^{{\rm{mCherry}}}$$

(obtained from cells transfected with pmCherry-C1). To obtain the final mean fluorescence values (F), the signal from cells untreated with inhibitor was subtracted. The data were obtained in triplicates, and half-maximal effective concentrations (EC_50_) were calculated using nonlinear regression analysis in GraphPad Prism v.7.03. (GraphPad Software). The error was expressed as a standard error.

#### Drug susceptibility

The drug susceptibilities of NL4-3 fully infectious virus was measured by determining the extent to which antiretroviral drugs inhibited the virus-induced cytopathic effect in MT-4 cells. Briefly, five-fold serial dilutions of antiretroviral drugs were added in triplicates in a 96-well plate with 30,000 MT-4 cells per well. After 1 h incubation, HIV was added at multiplicity of infection of 0.005 IU/cell. Following incubation at 37 °C with 5% CO_2_ for 5 days, the cell viability was determined by addition of XTT solution (Sigma-Aldrich) for 4 h, and the absorbance of newly formed orange formazan solution was measured using a Victor X3 plate reader (Perkin Elmer). Drug concentrations required to reduce the viral cytopathic effect by 50% (EC_50_) were calculated using GraphPad Prism v.7.03 (GraphPad Software) and used to determine the concentration range for combination studies.

#### Combination study

Evaluations of inhibitor combinations were performed in triplicates using the same method described above. Briefly, using constant ratio combination design in the range of 0.125x to 8x EC_50_, the effective dose was determined for darunavir, atazanavir and nelfinavir alone and in combination. The combination index (CI) at ED_50_, ED_75_, ED_90_, and ED_95_ was calculated using CompuSyn version 1.0.1. according to the Chou-Talay method^45^.

### Data availability statement

The datasets generated during and/or analyzed during the current study are available from the corresponding author on reasonable request.
